# A novel 3D pillar/well array platform using patient-derived head and neck tumor to predict the individual radioresponse

**DOI:** 10.1016/j.tranon.2022.101483

**Published:** 2022-07-16

**Authors:** Dong Woo Lee, Sung Yong Choi, Soo Yoon Kim, Hye Jin Kim, Da-Yong Shin, Joonho Shim, Bosung Ku, Dongryul Oh, Man Ki Chung

**Affiliations:** a Department of Biomedical Engineering, Gachon University, Republic of Korea; b Department of Otorhinolaryngology-Head and Neck Surgery, Uijeongbu Eulji Medical Center, Eulji University School of Medicine, Republic of Korea; c Department of Otorhinolaryngology-Head and Neck Surgery, Samsung Medical Center, Sungkyunkwan University School of Medicine, Seoul, Republic of Korea; d Department of Dermatology, Samsung Medical Center, Sungkyunkwan University School of Medicine, Seoul, Republic of Korea; e Central R & D Center, Medical & Bio Decision (MBD) Co., Ltd, Suwon, Republic of Korea; f Department of Radiation Oncology, Samsung Medical Center, Sungkyunkwan University School of Medicine, Seoul, Republic of Korea

**Keywords:** Head and neck neoplasm, Radiotherapy, Patient-derived cell, Precision medicine

## Abstract

Predicting individual radiotherapy (RT) response is valuable in managing head and neck squamous cell carcinoma (HNSCC). We assessed the feasibility of our novel 3D culture platform to measure radioresponse using patient-derived cells (PDCs) from HNSCC patients.

Cells from the FaDu line and tumor samples from 39 HNSCC patients were cultivated serially in Matrigel^TM^ on a 3D pillar/well array culture system. The 3D tumor models were exposed to 0 to 8 Gy of radiation dose, and the radioresponse index (RT_auc_, area under the dose-response curve) was measured quantitatively with Calcein AM staining of live tumor cells. Calcein AM fluorescence showed reduced density and the number of FaDu colonies as radiation increased, implying a dose-dependent effect on cell viability in the 3D pillar/well culture system. 3D tumor models using PDCs were established successfully from 39 HNSCC patient tumor samples, maintaining original genomic and pathological characteristics. These 3D tumor models were exposed to ionizing radiation on a 3D pillar/well array, with a mean period of 12 days from tumor harvest to the measurement of RT_auc_. The RT_auc_ of all PDCs varied from 3.5 to 9.4, and the lower 40th percentile (Z-score = -0.26) was considered a good radioresponse group with a threshold RT_auc_ of 4.6. The good radioresponse group showed fewer adverse features than others. As of the last follow-up, recurrence-free survival was better in the good radioresponse group (p = 0.037). 3D pillar/well array platforms using PDC could rapidly quantify radioresponse index in patients with HNSCC, showing potential as a novel prognosticator.

## Highlights

• Radiotherapy is a critical modality in head and neck cancer treatment.
• A novel 3D pillar/well array platform provides the individual radioresponse biomarker, RT_auc_.
• Poor and good radioresponse group by RT_auc_ correlates with other clinical features.
• RT_auc_ shows potential for radioresponse biomarker, useful in clinical decision-making.

## Introduction

Head and neck squamous cell carcinoma (HNSCC) is the seventh most common cancer, occurring in about 2,000 per 100,000 people every year globally. Its incidence has been increasing in recent years, even in young age groups, due to carcinogenesis caused by early tobacco consumption and human papillomavirus infection [1,2]. Approximately 40 to 60% of patients with HNSCC present with locally advanced disease, and its treatment remains challenging with current treatment resulting in 5-year survival rates of 30 to 40% [3,4]. In general, complete surgical resection is the backbone of the management of HNSCC. However, as surgical procedures often result in cosmetic and functional sequelae, which deteriorate the quality of life (QoL), treatment paradigms have shifted toward "organ preservation strategy", which focuses on non-surgical modalities such as radiation therapy (RT) with or without chemotherapy. In recent years, RT-based treatment has been preferred over radical surgery as a curative treatment of oropharyngeal, laryngeal, and hypopharyngeal cancers without compromising oncologic outcomes [5]. However, 20 to 40% of patients have locoregionally residual or recurrent tumors after RT. As a result, these poor radioresponse tumors require a salvage strategy, decreasing the probabilities of cure and good QoL.

In this regard, it is critical to select an appropriate group of patients for RT at the treatment decision-making stage to reduce the risk of treatment failure and improve the survival and QoL of HNSCC patients. Several studies have suggested various biomarkers to correlate with tumor radioresponse, i.e., multiple omics data, ultrasound radiomics, gene signatures, and clinicopathological predictors [6], [7], [8], [9]. Patient-derived culture (PDC) models (xenograft models, organoid models, or humanized mice models) recently have been shown to recapitulate the biological and genetic heterogeneity of patient-native tumors, offering a useful platform to test an individual tumor response to chemotherapeutic agents or irradiation [10]. Also, with the advancement of bioengineering techniques and microfluidics, the 3D culture method of PDCs has the advantages of being easily reproducible and applicable to a high-throughput platform while maintaining the diversity of patient-native cancer tissues [11,12]. Thus, it is widely applied to research on cancer mechanisms and responsiveness to various anticancer treatments [13], [14], [15], [16]. However, most previous studies regarding response to RT have been performed using tumor tissues stored in a biobank or grown in mice (PDX, patient-derived xenograft). These methods require a long time to obtain radioresponse outcomes, so it is challenging to use it at the treatment decision-making stage of clinical practice.

We previously investigated a 3D culture platform of PDC using a 3D pillar/well array system and demonstrated that it was helpful to quantify the invasive phenotype of HNSCC rapidly [17,18]. In the current study, we investigated the feasibility of our 3D culture platform to measure tumor radioresponse using 39 PDCs from HNSCC patients. Also, pathological and genetic profiles were compared against the original tumor to validate the 3D culture system. Finally, we evaluated the correlation of the radioresponse index with adverse clinicopathological features and oncologic outcomes.

## Materials and methods

### Patient-derived cell culture

The Institutional Review Board of Samsung Medical Center approved the acquisition of Patient-derived cell (PDC) samples and the relevant experimental protocol (SMC IRB file number 2015-06-132-008), and informed consent was obtained from all subjects. This work was performed in compliance with all relevant ethical regulations and guidelines for research using human specimens. PDCs were acquired from HNSCC patients to introduce a 3D culture in the pillar/well chip platform. The inclusion criteria of the present study were patients who (1) were over 18 years old; (2) had head and neck squamous cell carcinoma (HNSCC) confirmed by surgical pathology or biopsy; (3) had the proper integrity of medical records. Patients with a previous history of irradiation to the head and neck area or chemotherapy were excluded, however, recurrent HNSCC patients who underwent only surgical treatment were involved. Thus, among 39 patients, 35 patients had no previous treatments for HNSCC (fresh cases) and 4 patients were recurrent cases with previous surgery for HNSCC at the point of tumor collection. Detailed information on the HNSCC patients is presented in Table 1. Tissues from primary tumors were dissected into small pieces and washed with PBS. The minced tissue was digested with Minimum Essential Medium (MEM, Welgene, Daegu, Korea) containing DNase I (0.2 mg/mL, Sigma-Aldrich, MO, USA), dispase (4 mg/mL, Sigma-Aldrich, MO, USA), and collagenase (3 mg/mL, Sigma-Aldrich) at 37°C for 3 hr in a 37°C water bath to separate cells completely. After the addition of 10 mL of MEM with 10% fetal bovine serum (FBS, Gibco, Grand Island, NY, USA) and 1 × penicillin-streptomycin (P/S, Gibco, Grand Island, NY, USA), cell solutions were filtered using a cell strainer (70μm Nylon, FALCON, 352350, NY, USA) to remove unseparated tissue clusters. The cell solutions were centrifuged, and the pellet containing HNSCC cells was collected.

**Table 1:** Detailed information of the patients.

**No**	**RT_AUC_**	**Gender**	**Age**	**Primary site**	**Tx status**	**Stage**	**Treatment**	**Adjuvant Tx**	**HPV**	**RFS**	**Event**
1	5	F	78	FOM		pT3N2bM0	Surgery	CCRT		7	+
2	3.1	M	67	Hypophyarnx		pT3N3bM0	Surgery	CCRT		20	
3	3.2	M	51	Tongue		pT2N0M0	Surgery	.		18	
4	3.7	M	53	FOM		pT1N3bM0	Surgery	CCRT		1	
5	6	M	43	RMT		pT4aN0M0	Surgery	CCRT		1	
6	5.5	M	63	Tongue		pT2N0M0	Surgery	.		17	
7	4.4	F	58	FOM		pT1N2aM0	Surgery	CCRT		19	
8	3.9	M	63	Hypophyarnx		pT4aN1MO	Surgery	CCRT		18	
9	5.6	M	60	PNS		pT3N2bM0	Surgery	.		1	+
10	5.6	M	67	RMT		pT4aN0M0	Surgery	CCRT		17	
11	3.8	M	73	Cheek mucosa		pT2N0M0	Surgery	RT		12	+
12	6.3	F	69	Tongue		pT2N1M0	Surgery	CCRT		16	
13	4.3	F	58	Tonsil		pT1N1N0	Surgery	CCRT	positive	13	
14	6.8	M	61	RMT		pT1N0M0	Surgery	.		17	
15	4.7	F	76	FOM	s/p Surgery (recurrent)	rpT1N2bM0	Surgery	.		2	+
16	6.6	M	39	Tongue		pT3N2bM0	Surgery	CCRT		14	
17	4.8	M	36	FOM		pT3N0M0	Surgery	CCRT		13	
18	5.4	M	71	Tongue	s/p Surgery (recurrent)	rpT4aNxM0	Surgery	CCRT		7	+
19	6	M	60	PNS		pT4aN0M0	Surgery	RT		5	+
20	3.5	F	38	Tongue		pT3N0M0	Surgery	RT		54	
21	5.2	M	70	Tongue		pT4aN0M0	Surgery	RT		51	
22	5.2	M	50	PNS		pT3N2bM0	Surgery	.		1	+
23	3.3	M	81	FOM		pT2N0M0	Surgery	.		39	
24	4	F	54	PNS	s/p Surgery (recurrent)	rcT2N0M0	CCRT	.		5	+
25	3.2	M	63	Tonsil		cT2N1M0	CCRT	.	positive	28	
26	7.9	M	86	Tonsil		pT2NxM0	Surgery	.	negative	38	
27	6.7	M	73	Tonsil		cT2N1M0	CCRT	.	positive	35	
28	5.3	M	77	Tonsil		cT2N1M0	RT	.	positive	16	+
29	2.3	M	40	Tongue		pT2N0M0	Surgery	.		32	
30	4.8	M	78	Larynx	s/p Surgery (recurrent)	rpT4aN0M0	Surgery	.		31	+
31	9.4	M	73	Tongue		pT3N3bM0	Surgery	CCRT		35	
32	4	M	59	PNS		pT2N0M0	Surgery	RT		26	
33	3.1	M	76	Cheek mucosa		pT1N2M0	Surgery	RT		33	
34	6.5	M	72	Tonsil		cT2N0M0	RT	.	negative	27	
35	4.6	F	68	Tonsil		pT2N2M0	Surgery	CCRT	positive	31	
36	4.3	M	73	Hard palate		pT4aN1M0	Surgery	RT		32	
37	4.7	M	62	Tongue		pT3N1M0	Surgery	CCRT		18	+
38	6	F	79	PNS		pT3N0M0	Surgery	RT		4	+
39	4.9	M	69	Hypopharynx		cT3N2aM0	CCRT	.		9	+

Abbreviations: Tx, treatment, FOM, floor of mouth; RMT, retromolar trigone; PNS, paranasal sinus; RT, radiation therapy; CCRT, concurrent chemoradiation; RFS: recurrence-free survival (months), Event: recurrence or death, HPV, human papilloma virus.

### 3D tumor formation in the pillar/well array

The commercially available pillar/well array platform (Cellvitro™ 96PM, Medical & Bio Decision, South Korea) and its fabrication have been reported previously [19,20]. Before using the pillar/well array, the pillar array and cell-encapsulation apparatus were immersed in 70% ethanol for 30 min for sterilization, followed by complete drying at room temperature. Poly-L-Lysine (PLL) was coated on the surface of each pillar to increase adhesion between the pillar surface and the spheroid-containing Matrigel^TM^. A 2 µl mixture of PDCs and Matrigel^TM^ (Corning, NY, USA) was dispensed on the pillar surface by automatic spot dispenser (ASFA™ Spotter ST, Medical & Bio Decision, South Korea). A suspension of cells in Matrigel^TM^ was prepared by mixing equal volumes of cell suspension in F-medium (1 × 10^7^ cells/mL) with Matrigel^TM^ to obtain a final concentration of 5 × 10^6^ cells/mL and 50% alginate in the Matrigel^TM^. While changing cell seeding density, the final Matrigel^TM^ content remained constant at 50% throughout the experiment. The pillar array was inserted into a blank 96-well plate and placed on ice to prevent premature Matrigel^TM^ gelation. After 20 min, Matrigel^TM^ containing the aggregated cells was cross-linked and gelled by transferring the pillar array from ambient temperature to incubation at 37˚C for 15 min. The pillars were maintained upside down to allow cells to aggregate and form 3D tumors in the Matrigel^TM^. The 3D tumor models were cultivated in F medium [F-12 Nutrient Mixture (Sigma-Aldrich, MO, USA) and Dulbecco's modified Eagle's medium (Welgene, Daegu, Korea) (3:1), 5% fetal bovine serum (Gibco, Grand Island, NY, USA), 0.4 μg/mL hydrocortisone (Sigma-Aldrich, MO, USA), 5 μg/mL insulin (Sigma-Aldrich, MO, USA), 8.4 ng/mL cholera toxin (Sigma-Aldrich, MO, USA), 10 ng/mL epidermal growth factor (Invitrogen, CA, USA), 24 μg/mL adenine (Sigma-Aldrich, MO, USA), and 10 μmol/L Y-27632 (Enzo Life Science, USA)]. To evaluate the effects of radiation, cell viability exposed to radiation was compared with cell viability without radiation (baseline). Calcein AM stain is a conventional method to test the viable cells in 3D spheroids and organoids, and we already used Calcein AM in the clonogenic assay in pillar/well array [18]. Also, many previous works performed a drug screening assay using Calcein AM live-cell staining reagent in 3D cell culture-based drug screening platform [19,21,22].

To visualize tumor cells inside the Matrigel^TM^, we stained the pillar array containing 3D tumor models with 200 µL of 1 µM Calcein AM (Invitrogen, CA, USA) and 1 µg/mL of Hoechst 33258 dissolved in PBS buffer for 60 min in 96-well plates. Upon scanning, the green dots represented the cytosol of live cancer cells in the Matrigel^TM^, and blue dots indicated the nuclei of the attached cells on the pillar surface. The images were obtained at 4x magnification with a 490-nm excitation filter and a 520-nm emission filter under a fluorescent microscope (OLYMPUS BX51).

For histological analysis, PDCs (1 × 10^5/well) cells were seeded into round bottom ultra-low attachment 96well plate (corning costar, 7007). The cells were cultured in F media. After seven days, PDCs were formed spheroid, collected to 1.5ml tube with 1ml pipetman, and centrifuged. PDC cell pellets were fixed in 4% formaldehyde for 24 h at 4°C. After centrifugation (2 min, 1,300 rpm, RT), the supernatant was removed, and the cell pellet was embedded in paraffin (Histoplast PE paraffin; Epredia, USA) using an automated embedding system (Shandon Histocentre3, Thermo Electron Corporation, USA). For histochemistry, paraffin-embedded blocks were sectioned 4mm thick sections. Sections were used H&E staining or immunocytochemistry for cytokeratin (1:50, Abcam) and p53 (1:50, Leica).

For irradiation, cell pillar dishes were located under a 2 cm-thick bolus with a source surface distance of 100 cm and a field size of 30 × 30 cm^2^. Cells on the pillars were irradiated with 6-MV X-ray at single dose of 2, 4, 6 and 8 Gy in FaDu cells (purchased from ATCC, USA), and at two fractionated doses of 2, 4, 6 and 8 Gy for two consecutive days in patient-derived 3D tumors. For fractionated irradiation of PDCs, multiple pillar/well plates were prepared and the whole plate was irradiated with one radiation dose at each time. X-rays were delivered at a dose rate of 3.96 Gy per min using a Varian Clinac 6EX linear accelerator (Varian Medical Systems, Palo Alto, CA, USA). The absolute dose was calibrated according to TG-51 and verified using Gafchromic film to 1% accuracy. Irradiation were performed in triplicate for each dose.

### Image processing and data analysis

The 3D-cultured cells stained with Calcein AM were imaged by an automatic optical fluorescence scanner (ASFA™ Scanner ST, Medical & Bio Device, South Korea). The intensity of green fluorescence was measured using an 8-bit representation of each primary color. The viability of 3D-cultured cells was measured by quantifying the area of each green spot on the pillars. The area of each green spot was calculated by summing pixels in an area with an intensity higher than 20 code. Radiation response curves were obtained by plotting cell viability (total green area) according to radiation intensity (0, 2, 4,8 Gy) in GraphPad Prism 5 (Graph Pad Software, Inc.). The RT_auc_ was calculated automatically using XY analysis in GraphPad Prism 5.

### Immunofluorescence staining

For whole-mount staining of cells on pillars in the 96-well plate, cells were fixed with 4% paraformaldehyde (PFA) for 30 min on ice. After washing twice with PBS, samples were blocked with 5% goat serum in 0.5% Triton X-100 in PBS (PBST) for 60 min at room temperature. Cells on the pillars/well construct were incubated overnight at four°C with primary antibodies (diluted to a ratio of 1:200 with blocking solution). After several washes with PBS, samples were incubated for 2 h at RT with secondary antibodies and DAPI (diluted to a ratio of 1:500 with blocking solution). The following primary and secondary antibodies were used for immunostaining: anti-Ki-67 (rabbit monoclonal, SP6, Abcam) and anti-caspase-3 (rabbit polyclonal, 9661, Cell Signaling). FITC- or Cy3-conjugated secondary antibodies were purchased from Jackson ImmunoResearch. Nuclei were stained with DAPI (Invitrogen). Images were acquired using an LSM700 or LSM770 confocal microscope (Carl Zeiss).

### Targeted sequencing and variant detection

Genomic DNA was extracted from fresh tissue and 3D cultured cells using a QIAamp DNA mini kit (Qiagen, Valencia, CA, USA). In the case of 3D cultured cells, multiple pillar cultures were performed simultaneously to collect enough DNA amount for sequencing. DNA concentration and purity were quantified using a Nanodrop 8000 UV-Vis spectrometer (Thermo Fisher Scientific) and a Picogreen fluorescence assay using a Qubit 2.0 Fluorometer (Life Technologies). According to the manufacturer's instructions, the fragment size distribution was measured using a 2200 TapeStation Instrument (Agilent Technologies, Santa Clara, CA, USA). High-throughput sequencing was performed using CancerSCAN^TM^ panel version 3 containing 377 cancer-related genes as previously described (Table S1) [23,24]. Briefly, we used two published methods for detecting single-nucleotide variants (SNVs) with variant allele fraction (VAF) > 4%: MuTect v1.14 and LoFreq v0.61 [25,26]. Then, falsely detected variants from abnormally aligned, strand-biased, and clustered reads were filtered out using in-house scripts. Indels shorter than 30 bp were detected by Pindel [27]. Deletions of more than 30 bp and SVs were detected using JuLI [28]. The copy number of target genes was detected using an in-house copy number caller based on normalized copy number and B allele fractions of nearby SNPs.

### Statistical analysis

Prism software (Graph Pad Software, Inc.) was used to analyze the statistical significance of the comparisons by unpaired Student's t-tests or U-Mann Whitney tests. The Kaplan-Meier estimates and the log-rank test were used to assess the equality of survival functions across variables in the survival analysis. All statistical tests were two-sided, and significance was defined as *P <* 0.05.

## Results

### 3D pillar/well array system as a platform for radioresponse screening

**Fig. 1:**
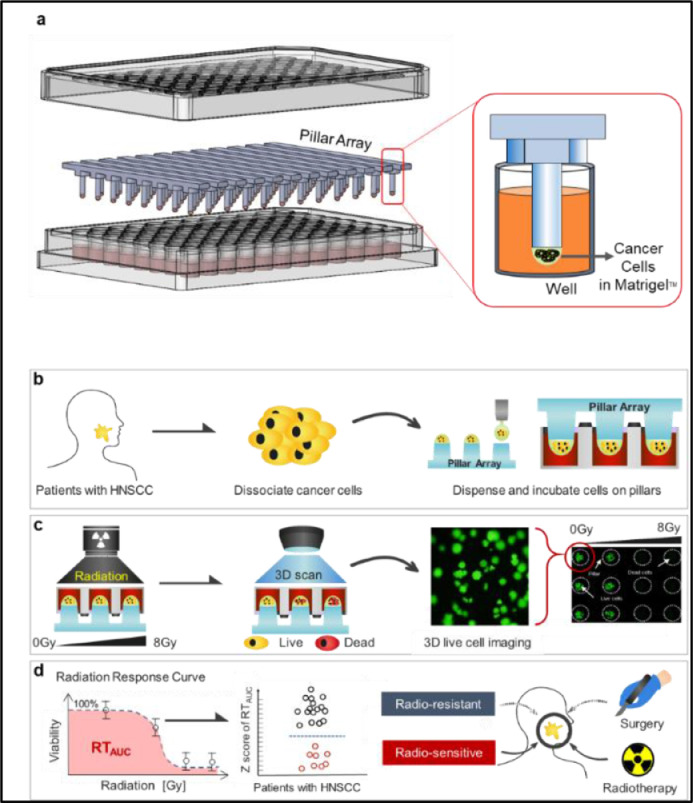
Graphical summary of the experimental design using the 3D pillar/well array system. **a** Pillar/well array containing eight pillars, each measuring 2 mm in diameter, 9 mm in height, and 9 mm in distance between pillars in conventional 96-well plates. By upside-down positioning of the pillar within the culture media in the well, cancer cells on the pillar were 3-dimensionally aggregated within the Matrigel^TM^ spot (red box). **b** Schematic diagram depicting a series of procedures for use of the 3D pillar/well culture system for head and neck cancer patients. **c** Schematic diagram depicting the irradiation process and scanning for 3D live cell imaging by Calcein AM staining. **d** Schematic outline of the therapeutic decision-making process based on the radiation response curve (RT_auc_) measured using the 3D pillar/well array system.

To develop a new radioresponse screening model that reflects the physiology of human cancer, we adapted a 3D patient-derived culture system using a pillar/well chip (3D pillar array system). As illustrated in Fig. 1a, a single pillar array containing eight pillars (2 mm in diameter, 9 mm in height, and 9 mm between pillars) was compatible with the conventional 96-well plate. Of note, upside-down culturing of the cancer cells within the Matrigel™ mixture with growth media-induced naturally aggregated cells on each pillar, which indicates that the 3D tumor formation process mimicked the physiology of solid tumors in humans (Figs. 1b and Supplementary 1). Therefore, we tested if we could recapitulate the response to radiotherapy in our 3D pillar/well array system and investigated whether the platforms could be used to predict radiation response in patient tumor models within two weeks, the time frame to help clinicians choose a more informed treatment approach for head and neck cancer. The 3D tumor models on a 3D pillar/well array system were exposed to fractionated radiation intensities of 0, 2, 4, 6, and 8 Gy. Then, viable tumors were stained with Calcein AM and quantitatively measured with an automated program. The area under the viable tumor plot (RT_auc_) curve was used as a representative value of radioresponse. Ideally, in clinical practice, this information on radioresponse would be made available during the initial staging workup, and patients with 'good radioresponse tumor' would be guided to receive radiotherapy (Fig. 1c and d).

### FaDu cell lines show dose-dependent responsiveness to radiation in the 3D pillar/well array system

**Fig. 2:**
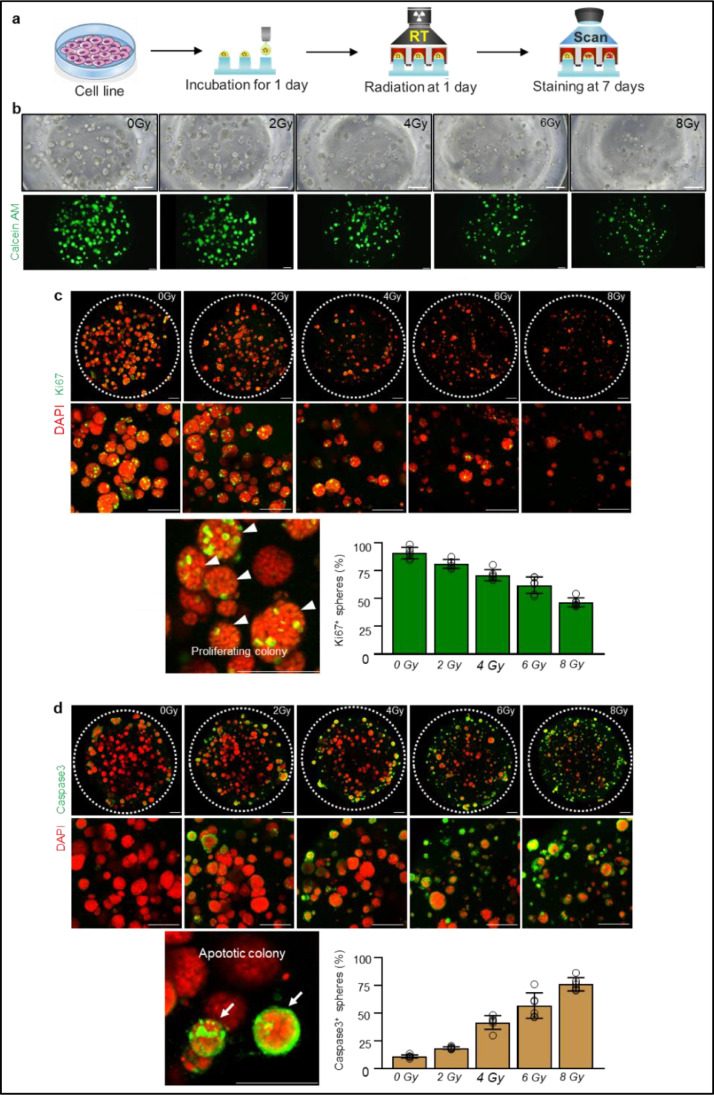
FaDu cell lines show dose-dependent responsiveness to radiation in the present 3D pillar/well array system. (**a)** Schematic diagram depicting the experimental schedules. (**b)** Brightfield images and Calcein AM staining of 3D tumors plated in the 94-well cell culture plates for radioresponse screening. Scale bars, 500 μm. (**c)** Representative images and comparison with the Ki67^+^ proliferating colony (white arrowhead) for each radiation dose. Scale bars, 500 μm. (**d)** Representative images and comparison with the Caspase3^+^ apoptotic colony (white arrow) for each radiation dose. Scale bars, 500 μm.

To validate the potential of the 3D pillar/well array system for radioresponse screening, we first investigated the FaDu cell line, a typical HNSCC cell line. Briefly, in this system, colonies on each pillar were confirmed four days after incubation and then irradiated with a single fraction of 0–8 Gy using a linear accelerator. Calcein AM fluorescence showed reduced density and the number of FaDu colonies as radiation increased (Fig. 2a), implying a dose-dependent effect of radiation on cell viability in the 3D pillar/well culture system. Next, to assess the biological effects of radiation on FaDu cells, we performed confocal microscopic analysis using whole-mount immunofluorescence staining. Compared with the control group (0 Gy), radiation reduced the number of proliferating colonies by 11, 22, 32, and 48% in the 2, 4, 6, and 8 Gy groups, respectively (Fig. 2b). Furthermore, the number of apoptotic colonies markedly increased by 80, 310, 460, 660% following each respective radiation dose (Fig. 2c), confirming that radiation exposure provokes DNA damage and leads to cellular senescence or apoptosis.

### Establishment of patient-derived cancer culture (PDC) on the 3D pillar/well array system

**Fig. 3:**
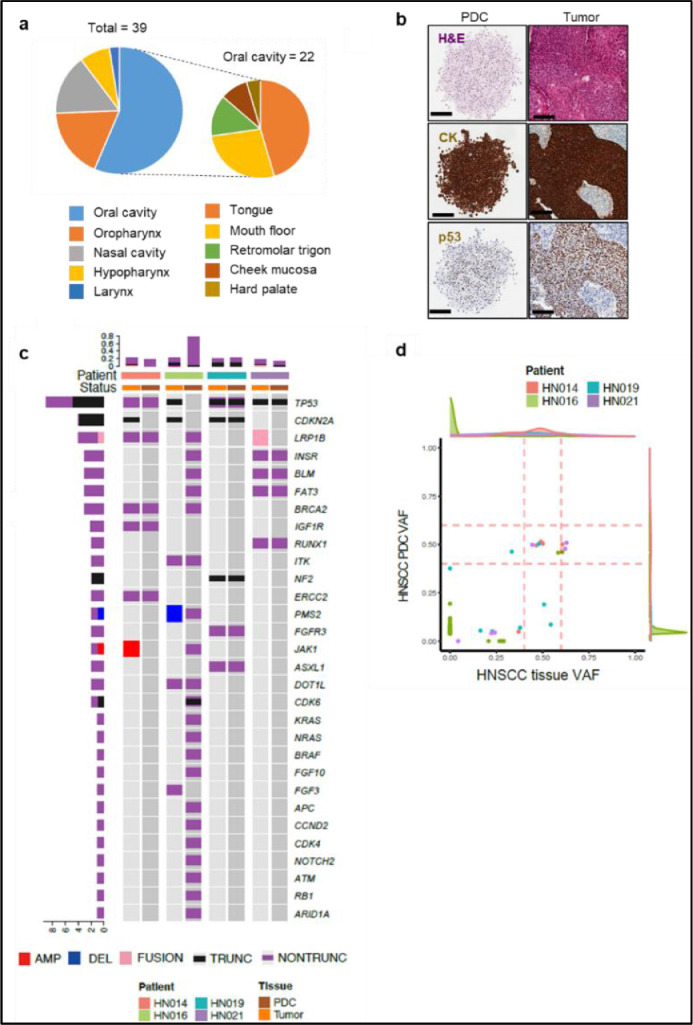
The 3D pillar/well array system maintains the genetic characteristics of the original tissues. (**a**) Overview and anatomic location of the cancers of which 3D tumors were established in the 3D pillar/well array system. (**b**) Hematoxylin and eosin (H&E) staining and immunostaining for tumor suppressor TP53 and pan-cytokeratin (CK) in paraffin-embedded 3D tumor models and their corresponding tissue (PDC, patient-derived cell). Scale bars, 100 μm. (**c**) Mutations were detected in 3D tumors on the pillar and their corresponding tissues, which were sequenced using targeted sequencing. (**d**) Comparison of VAF of genetic alterations detected in 3D tumor models and their corresponding tissue.

We set out to grow 3D tumor models from patient-derived HNSCC tissues (Table 1) from the oral cavity (tongue, mouth floor, retromolar trigone, cheek, and hard palate), oropharynx (tonsil), hypopharynx, larynx, and nasal cavity (Fig. 3a). Notably, we attempted to induce direct 3D tumor formation on the pillars without a cell expansion process not to increase the efficiency of our pillar system but to reduce the effect of *in vitro* selections or mutations. The direct 3D tumor formation system works well. Moreover, it shows a high success rate (39/42, 92.8%), suggesting that the 3D pillar/well system is a more feasible and practical tool for the analysis of radioresponse testing in the context of cancer biology.

Next, to determine if the direct PDCs on the 3D pillar/well system maintain characteristics similar to their primary tumor, we compared the immunohistochemistry (IHC) staining of the original tumor with that of the corresponding PDC 3D tumors. Positive staining of pan-cytokeratin (CK) and TP53 was found in all cells of the PDCs and the epithelium of the corresponding tumor slide (Fig. 3b), indicating that PDCs retain histological characters similar to their original tumors.

To investigate whether PDC 3D tumor models maintain the genetic characteristics of their original tumors, we performed targeted sequencing, covering 377 cancer-related genes (Supplementary Table 1) [23]. By comparing somatic mutations in four primary tumors and their matched PDC 3D tumors, we observed that 19 somatic mutations (19/25; 76.0%) detected in the primary tumors were maintained in the matched 3D tumor models (Fig. 3c; Supplementary Table 2). Notably, HNSCC-associated genes, including *TP53* and *CDKN2A*, which were rarely detected in normal tissues, also were preserved in PDC 3D tumor models. Interestingly, PDC 3D models from the HN016 tumor showed a newly emerged subclone with a high mutation burden (Fig. 3d). The newly emerged subclone harbored multiple alterations affecting more than a single pathway, including RTK-RAS and cell cycle pathways (Fig. 3c; Supplementary Table 3) [29]. Consistent with our findings, a previous study on tumor organoids also reported that more mutations were observed at low VAF [30]. Taken together, these results demonstrated that our 3D pillar/well array system from patient-derived tumors recapitulated the histologic characteristics as well as the genomic status of the original tumor source and can be used for *in vitro* radioresponse screening.

### Patient-derived 3D tumor models show variable responsiveness to radiation in the 3D pillar/well assay system

**Fig. 4:**
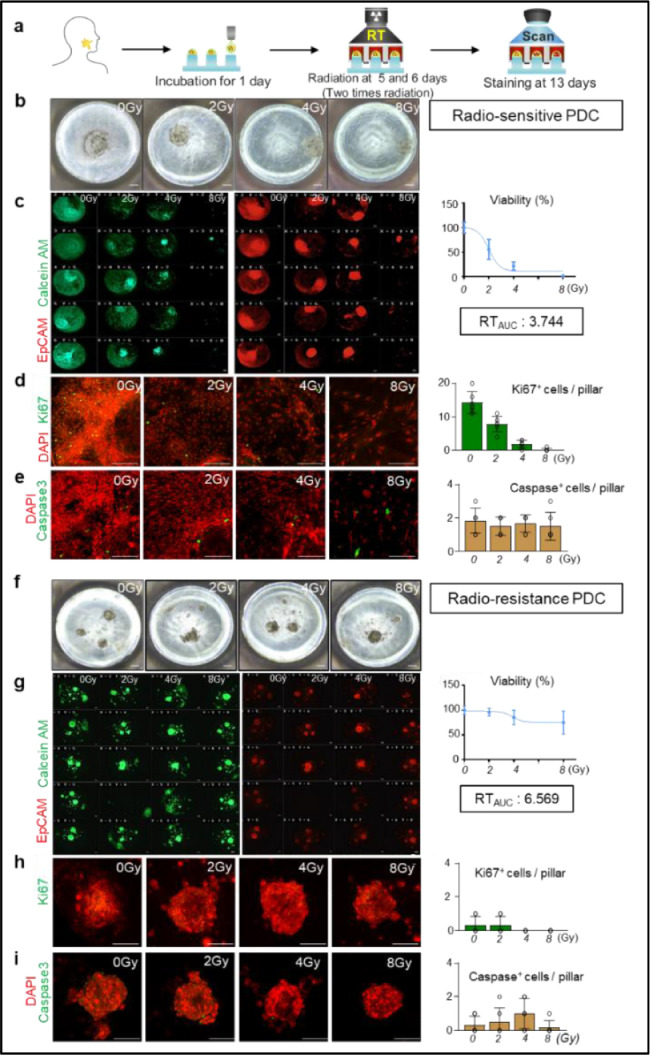
Radioresponse screening of 3D tumors from patient-derived tissues reveals heterogeneity of the radiation response. (**a**) Schematic diagram depicting the experimental schedules (**b**) Brightfield images of 3D tumor models from patient #4 in the good radioresponse group. Scale bars, 500 μm. (**c**) Analysis of RT_auc_ using 3D scan images for EpCam^+^ and Calcein^+^ 3D tumor models at each radiation dose. Scale bars, 500 μm. (**d**) Representative images and comparison of Ki67^+^ 3D tumor models at each radiation dose. Scale bars, 500 μm. (**e**) Representative images and comparison of Caspase3^+^ 3D tumor models at each radiation dose. Scale bars, 500 μm. (**f**) Brightfield images of 3D tumor models derived from patient #16 in the poor radioresponse group. Scale bars, 500 μm. (**g**) Analysis of RT_auc_ using 3D scan images for EpCam^+^ and Calcein^+^ 3D tumor models at each radiation dose. Scale bars, 500 μm. (**h**) Representative images and comparison of Ki67^+^ 3D tumor models at each radiation dose. Scale bars, 500 μm. (**i**) Representative images and comparison of Caspase3^+^ 3D tumor models at each radiation dose. Scale bars, 500 μm.

We exposed 39 HNSCC-derived 3D tumor models to ionizing radiation on a 3D pillar/well array and evaluated the radioresponse index by measuring a dose-response curve. The mean time period from primary tumor harvest to RT_auc_ measurement was 12 days (range, 10–12) (Fig. 4a). The RT_auc_ curves for all patients in this study were presented in Supplementary Fig. S2. As the response to radiation varied between PDCs, we calculated the Z-scores of RT_auc_ in all 39 cases as shown in Supplementary Fig. S3a. In this work, we compared RT_auc_ of patient-derived cells with the patient clinical response to RT. Based on optimal matching between clinical response and RT_auc_, we determined the cut-off value of RT_auc_ to predict radiation treatment. From 39 patients’ data, the lower 40th percentile (Z-score = -0.26) was considered a good radioresponse group with a threshold RT_auc_ of 4.6, and the true positive rate was 84.61% with this threshold (Supplementary Fig. S3b). Accordingly, patients were divided into two subgroups: good radioresponse group (less than 40th percentile of all RT_auc_ values) and the poor radioresponse group (more than 40th percentile of all RT_auc_ values).

Representative tumors of each group are illustrated in Fig. 4b–i. PDC 3D tumor models from patient #4 in the good radioresponse group showed higher responsiveness to radiation. Indeed, the number of double-positive PDCs for EpCam and Calcein decreased in a dose-dependent manner in patient #4, and the corresponding RT_auc_ value was 3.744 (Fig. 4b and c). The number of proliferating cells in a single pillar was reduced by 47, 87, and 98% in the 2, 4, and 8 Gy groups, respectively, while there was no difference in apoptotic cells among doses (Fig. 4d and e). Compared to good radioresponse PDC 3D tumor models, a poor radioresponse PDC tumor model from patient #16 was less responsive to radiation in the 3D pillar array, and its RT_auc_ value was 6.569 (Fig. 4f and g). Furthermore, there was no apparent difference in the number of double-positive PDCs, proliferating, or apoptotic cells among radiation doses (Fig. 4h and i), indicating significant interpatient variability in the PDC response to radiation.

### Correlation of the RT_auc_ biomarker with clinical outcomes

**Fig. 5:**
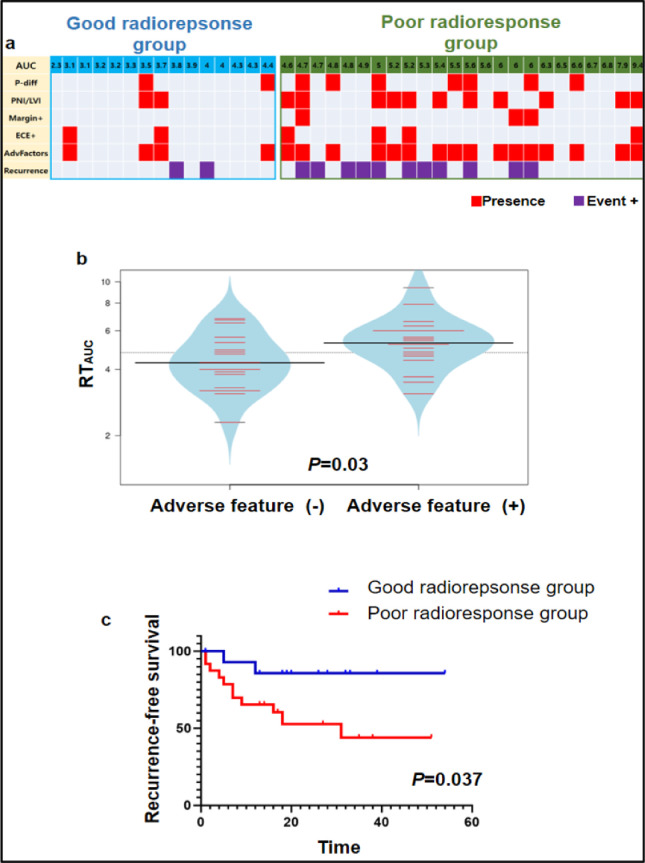
Correlation of radiation responses of 3D tumor models in the pillar/well array system with clinical outcomes in 39 patients. (**a**) 3D tumor model response data (RT_auc_) well match patient clinical outcomes (Recurrence) and adverse prognostic features such as poor differentiation (P-diff), perineural/lymphovascular invasion (PNI/LVI), margin involvement (Margin^+^), and extracapsular extension (ECE^+^). (**b**) Plot depicting RT_auc_ distribution for the adverse feature-negative group in comparison to the adverse factor-positive group (p = 0.03). (**c**) Kaplan-Meyer estimates of recurrence-free survival for the good radioresponse and poor radioresponse groups (p = 0.037).

To test whether the radioresponse biomarker (RT_auc_), measured using the 3D pillar/well array system, was clinically relevant for individual patients, we reviewed the retrospective clinical data from 39 HNSCC patients of the present study treated with curative intent. An overview of the clinical details is given in Table 1. Adverse clinicopathological features, including recurrence, survival status, distant metastasis, poor differentiation, and perineural/lymphovascular invasion, were more prevalent in the poor radioresponse group (Fig. 5a). The RT_auc_ of patients with adverse features was statistically higher than that of patients without adverse features (*P =* 0.03) (Fig. 5b). On survival analysis, patients with poor radioresponse 3D tumors showed significantly worse recurrence-free survival rates than patients with good radioresponse 3D tumors (*P =* 0.037) (Fig. 5c), suggesting the predictive potential of radioresponse screens using a 3D pillar spheroid array derived from HNSCC.

## Discussion

This study demonstrated the feasibility of a 3D pillar/well array system for rapid quantification of tumor radioresponse. First, it was shown that 3-dimensionally formed FaDu cell tumor models responded to radiation with increased Caspase3-positive cells and decreased Ki67-positive cells. Next, HNSCC patient-derived 3D tumor models in the pillar/well array presented different responses to radiation, classified as good radioresponse or poor radioresponse by a threshold RT_auc_ of 4.6. Finally, patients with higher RT_auc_ values (poor radioresponse) had more adverse clinicopathological features and recurrences, which suggests that RT_auc_ measured from a 3D pillar/well array system has the potential to be a novel prognosticator in HNSCC.

Predicting RT response is critical in therapeutic decision-making for the management of HNSCC. Many studies have investigated various ways to assess the radioresponse of individual patients, i.e., response to induction chemotherapy, functional imaging modalities, and molecular markers; however, there has been no robust method that can be applied in actual clinical practice [31], [32], [33]. Several studies tested the feasibility of predicting treatment response through the use of 3D culture platforms [34], [35], [36]. Yao et al. established patient-derived organoids (PDO) from locally advanced rectal cancer samples stored in an organoid biobank [36]. They evaluated the response of PDO to chemotherapy and to RT and compared it with clinical response. Clinical responses were matched highly to PDO responses, with 84.43% accuracy, 78.01% sensitivity, and 91.97% specificity. Pasch et al. developed PDO from multiple histology results, including those of colorectal and pancreatic cancer and adenocarcinoma of the lung [34]. They measured the PDO response to chemotherapy and evaluated RT using an optical metabolic imaging method that refined individual cell metabolic activity alongside size measurement. Additionally, they demonstrated one clinical application of this technology in a patient with refractory metastatic colorectal cancer. A patient who previously failed the FOLFOX regimen (oxaliplatin, 5-FU, leucovorin) was re-treated with the regimen based on the intermediated response of the PDO. The patient showed a good clinical response and has maintained this response for more than one year.

The current study is different from previous reports of patient-derived culture models in several aspects. First, we aimed to develop a more clinically relevant culture model that can provide information about individual radioresponse. To meet this requirement, the period between tumor harvest and outcome measurement must be as short as possible, ideally within 2–3 weeks of the cancer workup (laboratory and imaging studies). As we collected tumors from 39 consecutive patients with HNSCC, it took an average of 5–6 days for patient-derived tumors to become stable within the Matrigel^TM^ on the pillars, two days for radiation, and 7-10 days for culturing, staining, and outcome measurement. If further defined, the clinician can use the radioresponse index measured from a 3D pillar/well array system to decide whether a patient should undergo upfront surgery or RT.

Second, the culture platform used in the present study is commercially available, and clinicians can apply the methods of this study to their practice. Also, the pillar/well array system is suitable for high-throughput and repetitive analysis with the aid of an automated tissue distributor and an image scanner. Lastly, Matrigel^TM^-based culture with the pillar/well array system could overcome the significant technical issues of previous patient-derived culture models. Many extracellular matrix-embedded 3D cell culture or organoid platforms have been applied to radioresponse measurement *in vitro,* in which model cells were grown in Matrigel^TM^ wells on the flat bottom of the well plate [34], [35], [36]. However, the shape of the Matrigel^TM^ spot in the aforementioned scheme might be uneven due to the surface condition and level of individual skill (Supplementary Fig. 1a). Also, it is challenging to form a 3D tumor spheroid if patient-derived cells do not have contact with each other within the Matrigel^TM^ spot. On the contrary, the pillar helps to form a uniform Matrigel^TM^ spot (2 ul) regardless of surface condition or experimenter skill, as shown in Supplementary Fig. 1b. Patient-derived cells on the pillar-attached Matrigel^TM^ spot gather and directly contact each other after turning the pillar upside down on the blank well at 4°C for 20 min. During the gelling of Matrigel^TM^, patient-derived cells have the opportunity to increase cell-to-cell interactions and form large colonies.

Another interesting finding of this study is the potential of the radioresponse index as a novel prognosticator. When the cohort of this study was divided into good radioresponse and poor radioresponse groups by a threshold of AUC 4.6, the poor radioresponse group showed worse clinicopathological characteristics and tended to have a more frequent recurrence. This finding indicates that radioresponse measurement can be used as a prognostic factor in patients with HNSCC. Further research is needed to validate this finding.

The clonogenic assay is a widely used *in vitro* cell survival assay based on the ability of a single cell to grow into a colony. Thus, cell lines or organoids, which are derived from stem cell-containing tissue, are usually employed to test their clonogenic abilities for the effects of drugs or radiations on the growth and self-renewal potential of stem cells as well as their progenitor. However, in our 3D tumor model, we directly induced 3D tumor formation from patient-derived HNSCC tissues without expansion for making cell lines or selection for cancer stem cells, which indicates that our system is not amenable to forming colonies *in vitro*. We adopted this methodology to expedite the time period from tissue acquisition to radiation sensitivity measurement.

Also, quantitation of γ-H2AX foci has been applied as a useful tool for the evaluation of the efficacy of radiation because phosphorylation of histone H2AX to form γ-H2AX is a known marker for irradiation-induced DNA damages [37,38]. Although γ-H2AX assay was not applied in this study, we used cleaved-caspase 3 staining for detecting apoptotic cells in Figs. 2 and 3. Recently, Gionchiigla et al. demonstrated that many γH2AX immune-reactive irradiated cells in brain undergo apoptosis with cleavage of caspase 3 [39]. Another study also revealed that caspase 3 pathway is required for H2AX phosphorylation and apoptosis, suggesting that our analysis using caspase 3 could be an indirect surrogate for irradiation-induced DNA damages and apoptosis [40]. As Calcein AM is used for live-cell staining, Calcein AM fluorescence was generally reduced as radiation dose and irradiation-induced apoptosis increased. Therefore, previous experimental systems for radiosensitivity screening showed the inverse correlation between Calcein AM and γ-H2AX analysis. Thus, our system based on Calcein AM staining could be a feasible and acceptable tool for the analysis of radiosensitivity screening.

The cohort we used in this study was treated mainly by upfront surgery (36/39, 92.3%), and tumors were harvested from surgically resected samples. Although 63.8% (23/36) of the surgery group received adjuvant RT after surgery, it would have been more appropriate to interpret the RT_auc_ in radioresponse if most of the patients were treated with definitive RT first. We chose surgically-treated patients because it was easier to obtain a large tumor volume for the successful culture of primary tissues than to do so using small biopsies from RT patients.

In conclusion, this study demonstrated that individual radioresponse of HNSCC could be quantified by RT_auc_ values derived from the patient-derived 3D tumor pillar/well culture array system early enough to inform treatment decision-making in clinical practice.

## Funding

This work was supported by the National Research Foundation of Korea (NRF) grant funded by the Korea government (No. NRF-2020M2D9A3094087, P.I. Dongryul Oh) (No. NRF 2018R1D1A1B07044974, P.I. SY Choi) (No. 2019R1F1A1057795, P.I. MK Chung) and the Korea Medical Device Development Fund grant funded by the Korea government (the Ministry of Science and ICT, the Ministry of Trade, Industry and Energy, the Ministry of Health & Welfare, the Ministry of Food and Drug Safety) (Project Number: KMDF_PR_20200901_0135-2021)

## Informed consent statement

Informed consent was obtained from all the patients in the current study.

## Data availability statement

Datasets generated and analyzed during the current study are available from the corresponding author on reasonable request.

## Supplementary materials

Supplementary Fig. S1. Schematic view of (a) conventional ECM-embedded 3D cell culture and (b) Pillar/well-based ECM-embedded 3D cell culture.

Supplementary Fig. S2. The RT_auc_ curves for all patients.

Supplementary Fig. S3. (a) RT_auc_ of study patients shows the standard normal distribution, and a Z-score below -0.26 (corresponding to RT_auc_ under 40%) was defined as a good radioresponse group. (b) Plot depicting RT_auc_ Z-scores below -0.26 from 39 patients.

Supplementary Table S1.

Supplementary Table S2.

Supplementary Table S3.

## CRediT authorship contribution statement

**Dong Woo Lee:** Conceptualization, Methodology, Resources, Data curation, Writing – original draft, Funding acquisition. **Sung Yong Choi:** Conceptualization, Methodology, Data curation, Writing – original draft, Funding acquisition. **Soo Yoon Kim:** Investigation, Data curation. **Hye Jin Kim:** Investigation, Data curation. **Da-Yong Shin:** . **Joonho Shim:** Investigation, Data curation. **Bosung Ku:** Investigation, Resources. **Dongryul Oh:** Conceptualization, Methodology, Resources, Writing – review & editing, Funding acquisition. **Man Ki Chung:** Conceptualization, Methodology, Resources, Writing – review & editing, Funding acquisition.

## Declaration of Competing Interest

The authors declare that they have no conflict of interest.

## Appendix Supplementary materials

Image, application 1

Image, application 2

Image, application 3

Image, application 4
