# Supplementary material for: A novel 3D pillar/well array platform using patient-derived head and neck tumor to predict the individual radioresponse
Source: Transl Oncol. 2022 Jul 16;24:101483. doi: 10.1016/j.tranon.2022.101483 (PMC9294182; doi:10.1016/j.tranon.2022.101483)
Supplement: Supplementary file 1 [file mmc1.pptx]

## Slide 1
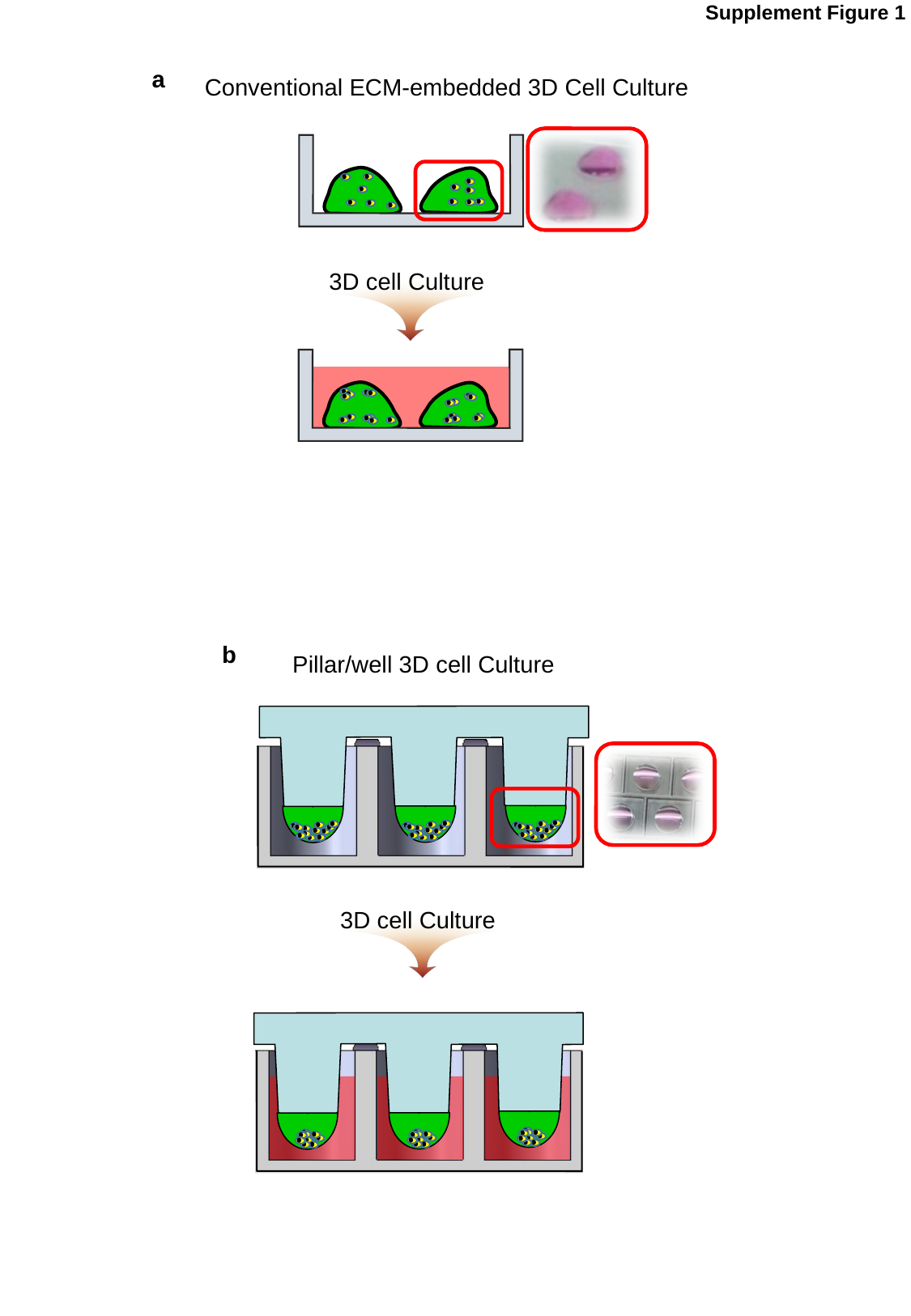

Supplement Figure 1
Conventional ECM-embedded 3D Cell Culture
a
3D cell Culture
b
 Pillar/well 3D cell Culture
3D cell Culture

## Slide 2
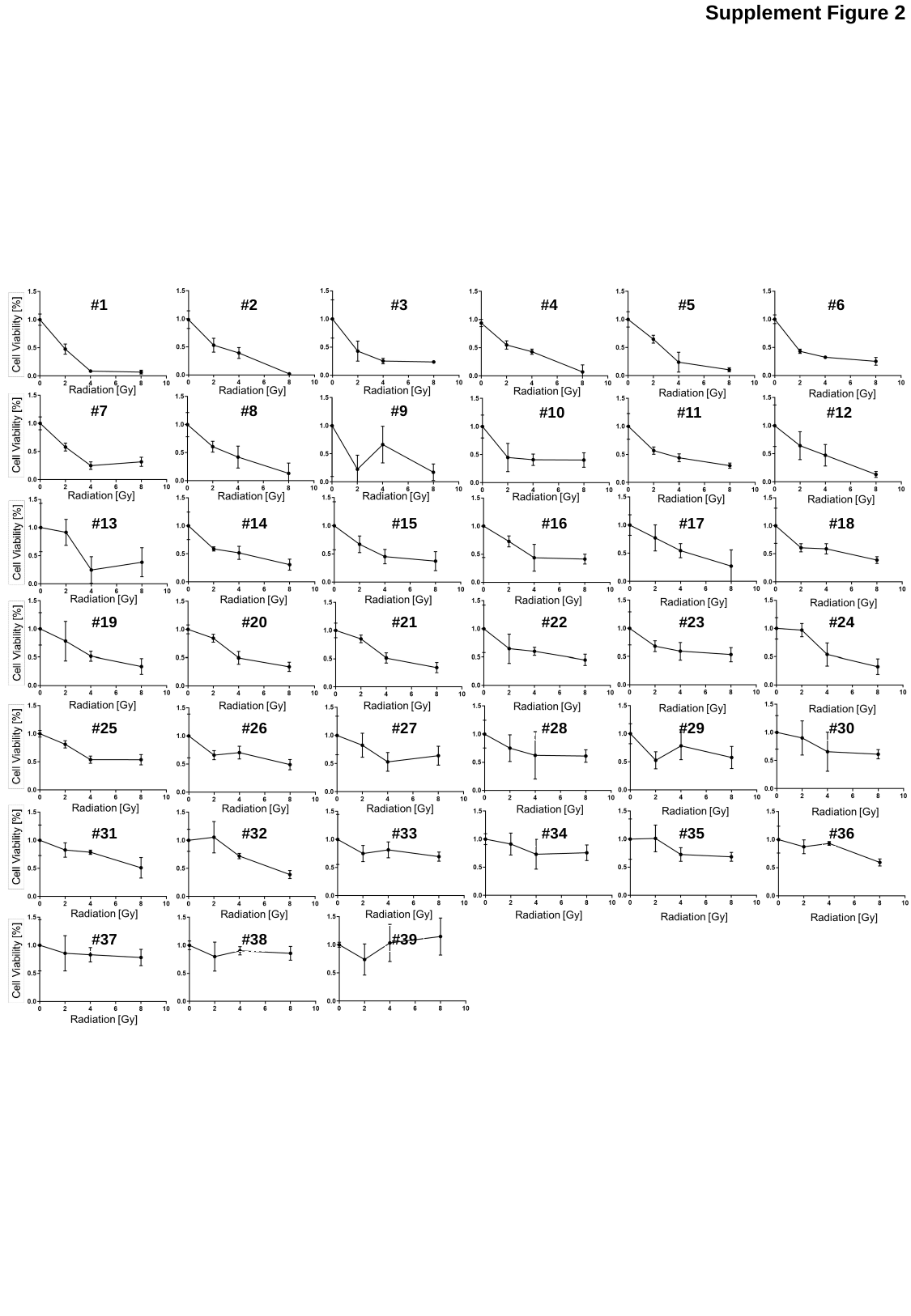

Supplement Figure 2
#1
#2
#3
#4
#5
#6
#10
#11
#12
#7
#8
#9
#13
#14
#15
#16
#17
#18
#19
#20
#21
#22
#23
#24
#25
#26
#27
#28
#29
#30
#31
#32
#33
#34
#35
#36
#37
#38
#39

## Slide 3
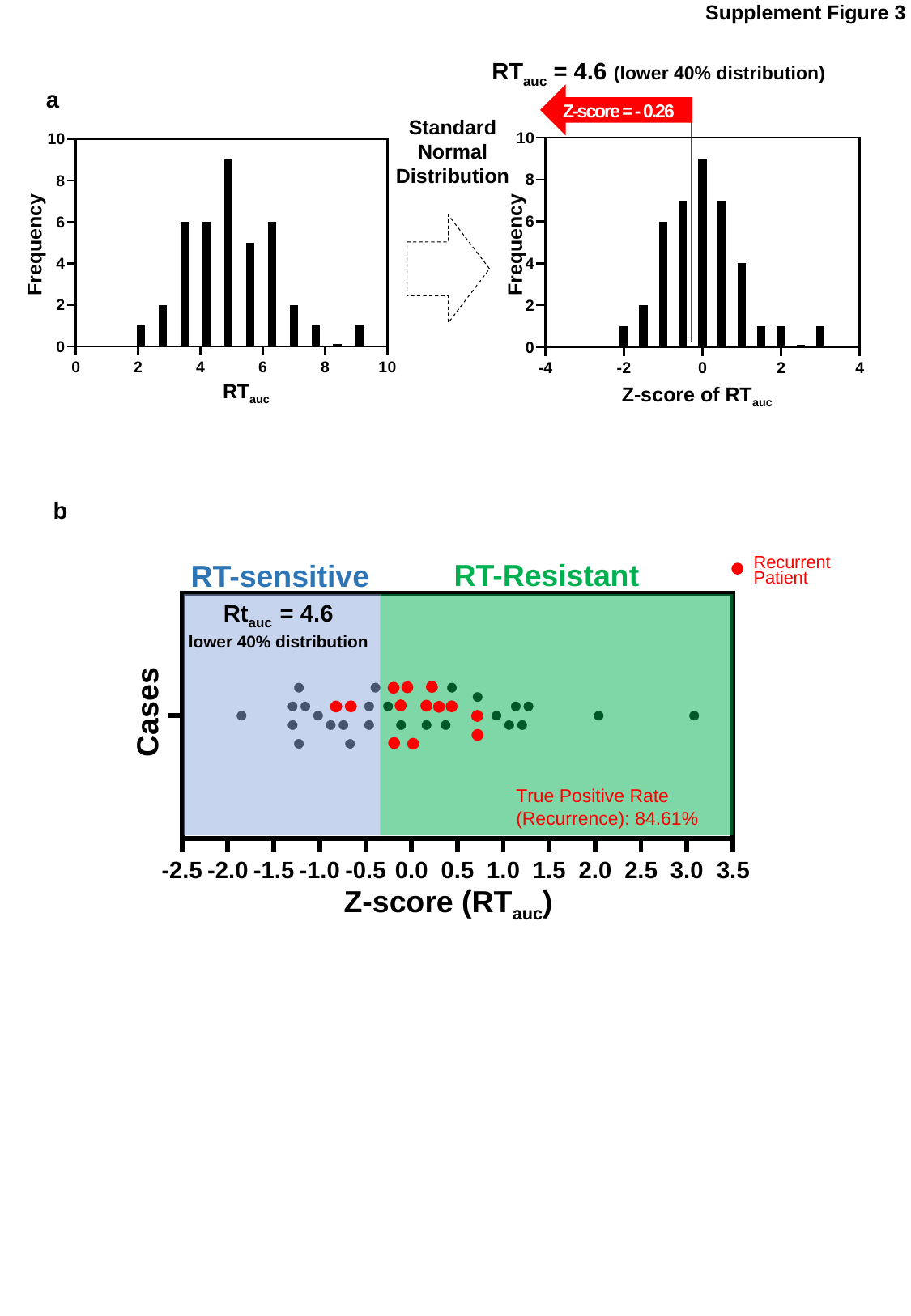

Supplement Figure 3
RTauc = 4.6 (lower 40% distribution)
a
Z-score = - 0.26
Standard Normal Distribution
Frequency
Frequency
RTauc
Z-score of RTauc
b
Z-score (RTauc)
RT-Resistant
RT-sensitive
Rtauc = 4.6
lower 40% distribution
Cases
Recurrent
Patient
True Positive Rate
(Recurrence): 84.61%
